# A Text-Book on Surgery

**Published:** 1898-09

**Authors:** 


A TEXT-BOOK ON SURGERY. General, Operative and Mechanical. By JOHN A. WYETH, M. D., Professor of Surgery in and President of the Faculty of the New York Polyclinic Medical School and Hospital; State Surgeon to Mount Sinai Hospital and Consulting Surgeon to St. Elizabeth's Hospital; Member of the New York Pathological Society, of the New York Surgical Society, etc., etc. Third edition, revised and enlarged. Royal octavo, pp. 997. New York: D. Appleton & Co. 1898.

This, the third edition of Dr. Wyeth's very excellent and comprehensive work at first glance is disappointing, though through no fault of the author. In these days of up-to-dativeness one expects progress in book making as in everything else and the very fact that the immense strides made in surgery within the past seven years, or since the second edition of this work was published, have made this enlarged and revised volume necessary, would naturally lead one to expect the publishers to do their share toward keeping up with the onward movement.

The illustrations in this volume are disappointing to the modern student, especially so as he has learned that illustrations are in many cases the most comprehensive portions of later scientific works. There is a befo'-the-war flavor about some of the illustrations in

Wyeth's surgery which stamps them as the handiwork of men who belonged to the wood-cut era of book illustration and which does not go well with the excellence and grace of the text, which has been brought right up to the present moment.

Messrs. D. Appleton & Co. can do better work. They have shown it in their publication of Dr. Kelly's work on gynecology, and the surgery of Dr. Wyeth certainly deserves better illustrations. Really, in looking at some of the figures one can almost see the swamps of the South; one almost expects to see some one of the stricken figures turn wearily and ask from his bed of pain: "What's new from Grant?" so typical of the 1860-70 era are the hang of the clothes, the cut of the hair and the trim of the beard of the figures. True, there are some new illustrations, but they are too few. There are a couple or so of skiagraphic reproductions and there are some very excellent and instructive illustrations in the section devoted to surgery of the stomach and intestines. These illustrations are taken from and credited to Park's System of Surgery.

The body of the work is just exactly what one would expect from the pen of such a past-master of the scalpel as Wyeth and can be made excellent use of by the general practitioner who is liable at any time to be called upon to operate. Wyeth has a faculty of presenting himself and making himself understood, which is rare among latter-day writers. The book is not fitted for the specialist, nor is it apparently intended for him. It is just what it claims to be—a plain, common sense, every-day treatise on surgery, taking in "non-infective and infective inflammations," the process of repair; specific and nonspecific urethritis; the surgical diseases, minor surgery, asepsis and antisepsis; amputations, fractures, surgery of the lymphatics and arteries, surgical diseases of the bones and joints; regional surgery: a brief chapter on deformities and an altogether too brief chapter on tumors.

The chapters on amputations deserve most flattering commendation. The methods and manners and procedures are presented in a way which is instructive and entertaining and the illustrations do credit to this portion of the book at least. Dr. Wyeth has described the various amputating operations so carefully and in so masterly a manner that a practitioner who erred after a careful study of the work could hardly be excused.

The field of general surgery has been amply covered within the past five years in the numerous volumes published. Park just about cleaned up everything in sight and made general works unnecessary when he published his system. The bent in surgical literature from now on should be monographical; special works on special subjects in surgery, dealing with but one subject between the two book covers and dealing with that one thoroughly. There are enough systems of surgery now published to keep the world informed for many years to come. There may be some new methods discovered, some new operations devised; but to make them the basis of a system of surgery would necessitate the rewriting of some one else's system and a resultant literary hash, which no matter how highly seasoned would, nevertheless, be hash, even though it were topped with a new idea.

